# Prevalence and risk factors of diabetes in a large community-based study in the Sultanate of Oman: STEPS survey 2017

**DOI:** 10.1186/s12902-020-00655-9

**Published:** 2021-03-05

**Authors:** Adhra Al-Mawali, Ayaman Al-Harrasi, Sathish Kumar Jayapal, Magdi Morsi, Avinash Daniel Pinto, Waleed Al-Shekaili, Hilal Al-Kharusi, Zainab Al-Balushi, John Idikula

**Affiliations:** 1 Centre of Studies & Research, Ministry of Health, Muscat, Sultanate of Oman; 2 Strategic Research Program for Non-Communicable Diseases, Ministry of Higher Education, Research and Innovation, Muscat, Sultanate of Oman

**Keywords:** Diabetes mellitus, Prevalence, Risk factors, Diabetes, Prediabetes, Oman, STEPS, NCD, Non-communicable disease, Control status

## Abstract

**Background:**

Type 2 diabetes in the Gulf Cooperation Council countries, including Oman, is currently the fastest growing health crisis and is a significant cause of premature mortality and disability. There is currently insufficient up-to-date information available on prevalence of type 2 diabetes. This study aimed to assess the latest prevalence of type 2 diabetes mellitus and its associated demographic, behavioural, and clinical risk factors.

**Methods:**

Using the WHO STEPwise approach to chronic disease surveillance, a nationally representative population-based survey was conducted from January to April 2017 of adults aged 18 years and above. A multi-stage, stratified, geographically clustered random sampling surveyed 9053 households including Omani nationals and non-Omani residents. Univariate and multiple logistic regression analysis was performed to determine the predictors of diabetes.

**Results:**

Overall prevalence of diabetes among the population was 15.7% (95% CI: 14.0–17.5%) whereas prevalence of prediabetes was 11.8% (95% CI: 11.4–12.2%). Age, educational level, raised blood pressure, family history of diabetes, abnormal waist-to-hip ratio, and hypertriglyceridemia were found to be significantly associated with diabetes mellitus. Of the cases of diabetes mellitus, 17% were newly diagnosed and 13.2% were on medication and had an uncontrolled glucose level while 55.5% were not taking medication (although diagnosed) and had an uncontrolled blood glucose level.

**Conclusions:**

The present study provides reliable information regarding the high prevalence of diabetes mellitus among the adult population in Oman with urgent attention needed to address this significant burden on the health system. The high proportion of uncontrolled cases warrants further research, awareness programmes, and community interventions.

## Background

Around 463 million people had diabetes mellitus (DM) in 2019 based on International Diabetes Federation estimates, with a projection of 700 million by 2040. From this, it is evident that DM is one of the fastest growing health crises facing the twenty-first century. Type 2 diabetes, the most common form, accounts for about 90% of all global diabetes cases. The global economic burden of DM is massive, with an estimated annual expenditure of USD 760 billion in 2019, projected to grow by 11.2% to USD 845 billion by 2045 [1].

Recent literature revealed that the prevalence of type 2 diabetes in the Gulf Cooperation Council (GCC) is high with a dramatic increase in the past two decades, and is expected to increase by 96.5% by 2045 [2, 3]. Oman has been classified by the World Health Organization (WHO) as a country with low child and adult mortality rates (crude death rate: 2.4 per 1000). However, DM is currently the sixth leading cause of premature mortality in Oman and the fifth prominent cause of disability-adjusted life years lost [4].

Diabetes prevalence estimation and identification of high-risk groups is vital for surveillance, and development of policies and community-based interventions. There is currently insufficient, up-to-date information available on prevalence of type 2 diabetes and its correlates in the Sultanate of Oman. Previous community-based studies in Oman have been limited and do not truly represent all the risk factors and its relationships with diabetes in the Oman population [5–7]. This survey study, comprising the largest population-based survey in Oman so far, in collaboration with the WHO aimed to assess the latest prevalence of type 2 DM and its associated demographic, behavioural, and clinical risk factors.

## Methods

### Sampling

Briefly, and as previously described in the STEPS survey 2017 main paper [8], a nation-wide non-communicable disease (NCD) risk factor survey based on WHO STEPwise approach to chronic disease surveillance was undertaken in all the governorates (regions) in the Sultanate of Oman from January 2017 to April 2017. The survey adopted a multi-stage stratified, geographically clustered random sampling approach using the 2010 national census sampling frame. Cluster sampling (based on the 2010 census blocks) was used and symmetric equal number of blocks were chosen in each governorate. A total of 9053 households, 823 households chosen from each governorate‚ were selected (Omani nationals and non-Omani national residents) with one adult being selected from each household randomly. Adjustment of sample weight was done for non-response at the household level. The target age group was 18 years or older.

### Data collection

A culturally adapted, Arabic translated, and pre-tested version of the WHO STEPS questionnaire (version 3.1) was used [8, 9]. As part of the household survey, sociodemographic and behavioural information on tobacco use, diet, physical activity, history of chronic diseases, family history of chronic conditions was collected in Step 1. Physical measurements such as height, weight, hip circumference, waist circumference, and blood pressure were collected in Step 2. Biochemical tests were conducted to measure fasting blood glucose, total cholesterol, and triglycerides in Step 3.

Data were collected by trained health professionals in the field. SECA adult portable stadiometers were used to measure height after removing shoes, socks, slippers and any head gear, with an accuracy up to 0.1 cm. SECA® digital weighing scales were used to measure the weight of the individuals. The scale was regularly calibrated against a standard weight. Waist circumference was measured using a SECA® constant tension tape to the nearest 0.1 cm. Blood pressure levels (both systolic blood pressure (SBP) and diastolic blood pressure (DBP)) were measured three times using the Omron M3 digital blood pressure device as recommended by WHO, with the average of the closest two readings taken as the final reading. Overnight fasting blood samples were drawn to assess the biochemical profile of participants. For blood glucose and lipid profile measurements, dry chemistry method was used (CardioChek® Plus Analyzer). The reference biomarker cut-off points are mentioned under the heading of operational definitions.

### Data analysis

Categorical variables are summarized using proportions and continuous variables using mean, with 95% confidence intervals. Chi square test was used for comparison of proportions across groups. Univariate and multiple logistic regression analysis was performed to determine the predictors of diabetes. All variables were entered into the multiple regression model to ensure that the interaction of all variables and their effects with the presence of other variables were measured. Statistical analysis was done using STATA/IC version 16.1.

### Ethical considerations

The survey was approved by the Central Research and Ethical Review & Approval Committee of the Ministry of Health, Sultanate of Oman. (Approval No: 26/2015). Written informed consent was obtained from participants for participation in the study separately during health history collection (STEP 1) and measurement of biophysiological parameters (STEP 2 & 3). The confidentiality of the data gathered was maintained through a secure server.

### Operational definitions

Diagnostic criteria were based on WHO guidelines where diabetes was defined as individuals diagnosed by a physician and/or on antidiabetic medications and/or those who had fasting blood glucose ≥7 mmol/l (≥126 mg/dl), while prediabetes or impaired fasting glucose was defined as fasting blood glucose ≥6.1 mmol/l (≥110 mg/dl) and < 7 mmol/l (< 126 mg/dl). Current smoking included those individuals who were reported smoking in the month preceding the survey. Raised blood pressure was defined as individuals diagnosed by a physician and on antihypertensive medications (self-reported) and/or those who had systolic blood pressure ≥ 140 mmHg and/or diastolic blood pressure ≥ 90 mmHg (WHO Criteria) [10, 11]. One serving or portion of vegetables was considered to be 80 g or 1 cup of raw green leafy vegetables or 1/2 cup of other cooked/chopped vegetables. One serving or portion of fruit was considered to be one whole medium-sized fruit or two smaller-sized fruits (WHO). Obesity was defined as Body Mass Index (BMI) ≥ 30. Hypertriglyceridemia was defined as serum triglyceride levels ≥150 mg/dl (≥1.7 mmol/l). Physical activity was assessed using the Global Physical Activity Questionnaire (GPAQ), which has been developed by the World Health Organization and used in the STEPS questionnaire [12, 13]. Participants were classified as sufficiently active when exceeding the minimum duration of physical activity per week recommended by WHO i.e. 150 min of moderate intensity physical activity or 75 min of vigorous intensity physical activity or an equivalent combination of moderate- and vigorous intensity physical activity [13]. Show cards (pictorial, adapted to the local context) were used to explain to the participants the type of physical activity, servings of fruits and vegetables and salty food intake [12].

## Results

Table 1 shows the demographic characteristics of the respondents in the study. The overall response rate among survey participants was 75.5%, with the figure for Omani nationals being 73.5%. Majority of respondents were 30–39 years old (32.4%), Omani nationals (70.6%), had completed secondary school (34.3%), and were not currently engaged in any form of employment (44.9%).

**Table 1:** Demographic characteristics of the participants

Characteristics	n	Percentage
**Age groups (years)**		
18–29	1647	24.4
30–39	2187	32.4
40–49	1410	20.9
50–59	708	10.5
60 and above	630	9.3
**Sex**		
Male	3365	51.1
Female	3217	48.9
**Nationality**		
Omani	4645	70.6
Non-Omani	1937	29.4
**Education Level**		
No formal education	1772	26.9
Preparatory or lower	988	15.0
Secondary completed	2258	34.3
University and above	1558	23.7
**Employment status**		
Public sector employee	1287	21.5
Private sector employee	1766	29.5
Self-employed	251	4.1
Not currently working	2692	44.9

### Prevalence of DM

15.7% (95% CI: 14.0–17.5%) of the survey participants were found to have DM (Table 2). The prevalence was similar among women (16.1%) and men (15.4%). A lower prevalence of raised blood glucose was seen in Omani nationals (14.5%) as compared to non-Omani residents (18.8%). Impaired glycaemia was prevalent among 11.8% of the population. Higher prevalence’s were observed among women (12.6%) and non-Omani residents (12.5%) as compared to men (11.1%,) and Omani (11.5%), respectively.

**Table 2:** Prevalence of diabetes mellitus (DM) and pre-diabetes by nationality and demographic characteristics

Characteristics	Omani		Non-Omani		Total	
	Prevalence of pre-diabetes N (%, 95 CI)	Prevalence of diabetes N (%, 95 CI)	Prevalence of pre-diabetes N (%, 95 CI)	Prevalence of diabetes N (%, 95 CI)	Prevalence of pre-diabetes N (%, 95 CI)	Prevalence of diabetes N (%, 95 CI)
**Age group**						
18–29	138 (8.6,(7.7–9.6))	55 (3.1,(2.1–4.4))	80 (11.8,(9.8–14.1))	34 (3.2,(1.9–5.2))	218 (9.3,(8.4–10.2))	89 (3.1,(2.3–4.2))
30–39	201 (12.8,(11.8–13.9))	119 (6.1,(4.7–7.8))	112 (13,(11.3–14.9))	99 (12.4,(9.2–16.5))	313 (12.9,(12.1–13.7))	218 (8.23,(6.7–10.0))
40–49	148 (14.6,(12.9–16.6))	166 (20.6,(14.7–28.1))	83 (11.3,(9.7–13))	105 (25.7,(20.0–32.2))	231 (13.5,(12.2–14.9))	271 (22.3,(17.9–27.5))
50–59	81 (10.5,(9.1–12))	162 (34.7,(26.6–3.7))	30 (15.3,(12.7–18.3))	81 (37.8,(27.5–49.3))	111 (11.7,(10.4–13))	243 (35.5,(28.8–42.8))
60 & above	91 (12.6,(11.5–13.9))	205 (39.8,(31.7–48.4))	5 (11.2,(7.4–16.5))	28 (58.0,(38.5–75.2))	96 (12.4,(11.3–13.6))	233 (42.6,(35.1–50.5))
**Sex**						
Male	249 (10.1,(9.4–10.8))	253 (13.5,(10.3–17.4))	261 (12.4,(11.1–13.9))	290 (18.4,(15.3–22.0))	510 (11,(10.3–11.7))	543 (15.4,(13.0–18.1))
Female	410 (12.6,(11.8–13.4))	454 (15.3,(13.0–18.1))	49 (12.3,(10.1–14.9))	57 (20.1,(14.3–27.5))	459 (12.6,(11.8–13.3))	511 (16.1,(13.8–18.6))
**Education level**						
No formal education	230 (15.5,(14.4–16.8))	373 (31.8,(27.0–37.0))	76 (14.7,(13.3–16.3))	83 (23.6,(17.5–30.9))	306 (15.4,(14.4–16.4))	456 (30.3,(26.2–34.8))
Preparatory or less	80 (12.6,(10.9–14.4))	98 (23.4,(17.6–30.3))	75 (13.5,(12.2–14.9))	88 (20.4,(15.4–26.5))	155 (12.9,(11.8–14.1))	186 (22.2,(18.1–27.0))
Secondary completed	224 (10.3,(9.6–11.1))	156 (8.5,(5.7–12.2))	74 (10.5,(8.6–12.7))	96 (22.3,(15.5–31.0))	298 (10.4,(9.7–11.1))	252 (11.0,(8.4–14.2))
University & above	124 (9.7,(8.6–11))	80 (7.57,(4.7–11.9))	85 (12.5,(10.6–14.6))	79 (15.0,(11.1–19.9))	209 (10.8,(9.8–11.9))	159 (10.5,(8.0–13.6))
**Marital Status**						
Never married	89 (8.3,(7.4–9.4))	42 (4.3,(2.1–8.5))	72 (12.1,(9.6–15.1))	33 (5.3,(2.8–9.8))	161 (9.1,(8.2–10.2))	75 (4.6,(2.6–7.7))
Currently married	496 (13,(12.3–13.7))	544 (17.3,(14.7–20.1))	233 (12.6,(11.3–13.9))	309 (22.2,(18.9–25.8))	729 (12.8,(12.3–13.4))	853 (18.8,(16.7–21.0))
Divorced/Separated	22 (12.3,(9.8–15.2))	91 (22.2,(8.8–45.5))	4 (16.2,(9.3–26.7))	2 (48.3,(12.5–85.9))	26 (12.5,(10.1–15.3))	103 (23.6,(10.4–5.3))
Widowed	52 (10.6,(9.1–12.3))	98 (34.7,(24.0–47.1))	1 (3.3,(1.7–6.2))	3 (30.5,(5.4–76.9))	53 (10.1,(8.7–11.7))	101 (34.4,(24.1–6.4))
**Work status**						
Public sector employee	163 (13.7,(12.3–15.1))	124 (11.4,(8.2–15.7))	32 (11.2,(8.7–14.2))	27 (13.6,(7.8–22.6))	195 (13.4,(12.2–14.7))	151 (11.6,(8.6–15.5))
Private sector employee	60 (5.9,(5.2–6.7))	77 (13.9,(7.5–24.4))	244 (12.5,(11.3–13.9))	278 (17.8,(14.7–21.3))	304 (10.3,(9.4–11.3))	355 (16.5,(13.3–20.3))
Not currently working	435 (11.9,(11.3–12.6))	506 (15.9,(13.5–18.5))	34 (12.4,(10.2–14.9))	42 (24.6,(17.0–34.2))	469 (11.9,(11.3–12.6))	548 (16.8,(14.5–19.4))
**Total**	659 (11.5,(11.0–12.1))	707 (14.5,(12.5–16.8))	310 (12.5,(11.7–13.3))	347 (18.8,(16.0–22.0))	969 (11.8,(11.4–12.2))	1054 (15.7,(14.0–17.5))

### Risk factors for DM

All demographic, lifestyle, and clinical variables were entered in a multivariate regression analysis model. In terms of demographic covariates, age group (*p* < .001) and educational level (*p* < .05) were found to be the risk factors significantly associated with DM (Table 3). In terms of lifestyle and clinical covariates, raised blood pressure (*p* < .05), family history of diabetes (*p* < .001), abnormal waist-to-hip ratio (*p* < .001), and hypertriglyceridemia (*p* < .001) were found to be significantly associated with DM (Table 4).

**Table 3:** Multivariate analysis for demographic covariates associated with type 2 diabetes

Covariate	Omani				Non-Omani				Total			
	Total N	Prevalence of diabetes n (%)	Odds Ratio (P(95% CI))	Multivariant Regression Analysis	Total N	Prevalence of diabetes N (%)	Odds Ratio (P(95% CI))	Multivariant Regression Analysis	Total N	Prevalence of diabetes n (%)	Odds Ratio (P(95% CI))	Multivariant Regression Analysis
				P(95% CI)				P(95% CI)				P(95% CI)
**Age group**				**0.000**				**0.000**				**0.000**
18–29	1102	55 (3.08)	Ref		437	34 (3.22)	Ref		1539	89 (3.1)	Ref	
30–39	1337	119 (6.11)	0.08 (0.778)		707	99 (12.44)	1.14**(0.001)		2044	218 (8.23)	0.48*(0.036)	
40–49	856	166 (20.59)	1.21***(0.000)		465	105 (25.66)	1.86***(0.000)		1321	271 (22.33)	1.40***(0.000)	
50–59	484	162 (34.69)	1.85***(0.000)		183	81 (37.8)	2.23***(0.000)		667	243 (35.48)	1.99***(0.000)	
60 and above	541	205 (39.76)	2.11***(0.000)		50	28 (57.96)	3.26***(0.000)		591	233 (42.64)	2.37***(0.000)	
**Sex**				**0.242**				**0.517**				**0.384**
Male	1665	253 (13.48)	Ref		1518	290 (18.41)	Ref		3183	543 (15.39)	Ref	
Female	2655	454 (15.38)	0.31 (0.148)		324	57 (20.11)	0.28 (0.500)		2979	511 (16.06)	0.33 (0.075)	
**Education level**				**0.048**				**0.003**				**0.022**
No formal education	1274	373 (31.77)	Ref		387	83 (23.61)	Ref		1661	456 (30.33)	Ref	
Preparatory or less	487	98 (23.38)	0.29 (0.244)		439	88 (20.41)	0.04 (0.878)		926	186 (22.22)	0.26 (0.186)	
Secondary completed	1648	156 (8.45)	−0.31 (0.253)		462	96 (22.34)	0.047 (0.865)		2110	252 (10.99)	−0.11 (0.597)	
University and above	908	80 (7.57)	−0.61 (0.073)		552	79 (14.97)	−0.80**(0.007)		1460	159 (10.48)	−0.48*(0.033)	
**Marital Status**				**0.977**				**0.561**				**0.835**
Never married	742	42 (4.34)	Ref		400	33 (5.32)	Ref		1378	75 (4.55)	Ref	
Currently married	3161	544 (17.25)	0.45 (0.145)		1422	309 (22.19)	0.57 (0.118)		4895	853 (18.8)	0.53*(0.035)	
Divorced/Separated	114	91 (22.17)	0.49 (0.427)		14	2 (48.25)	0.92 (0.244)		135	103 (23.63)	0.48 (0.390)	
Widowed	303	98 (34.68)	0.26 (0.541)		6	3 (30.47)	−0.54 (0.716)		335	101 (34.42)	0.23 (0.542)	
**Work status**				**0.371**				**0.459**				**0.532**
Public sector employee	1042	124 (11.41)	Ref		175	27 (13.58)	Ref		1217	151 (11.64)	Ref	
Private sector employee	465	77 (13.98)	0.09 (0.798)		1442	278 (17.79)	0.007 (0.986)		1907	355 (16.5)	0.28 (0.202)	
Not currently working	2810	506 (15.88)	−0.23 (0.454)		225	42 (24.63)	0.18 (0.708)		3035	548 (16.81)	−0.08 (0.743)	

*CI* Confidence interval; *p* values in parentheses, * *p* < 0.05 ** *p* < 0.01 *** *p* < 0.001

**Table 4:** Multivariate analysis for behavioural and clinical covariates associated with type 2 diabetes

	Omani				Non-Omani				Total			
	Total N	Diabetes n (%)	Odds Ratio (P(95% CI))	Multivariant Regression Analysis	Total N	Diabetes n (%)	Odds Ratio (P(95% CI))	Multivariant Regression Analysis	Total N	Diabetes n (%)	Odds Ratio (P(95% CI))	Multivariant Regression Analysis
				P(95% CI)				P(95% CI)				P(95% CI)
**Smoking**				**0.317**				**0.606**				**0.98**
Yes	224	38 (12.78)	Ref		302	66 (21.21)	Ref		526	104 (16.67)	Ref	
No	4096	669 (14.65)	−0.44 (0.223)		1540	281 (18.42)	0.12 (0.653)		5636	950 (15.61)	−0.07 (0.747)	
**Raised Blood pressure**^**a**^				**0.008**				**0.159**				**0.002**
No	2959	322 (8.65)	Ref		1233	185 (13.3)	Ref		4192	507 (9.84)	Ref	
Yes	1361	385 (26.84)	0.56**(0.004)		609	162 (28.14)	0.33 (0.133)		1970	547 (27.23)	0.51***(0.001)	
**≥ 5 servings of fruits and/or vegetable daily**				**0.561**				**0.554**				**0.868**
No	2706	447 (13.2)	Ref		1360	262 (18.43)	Ref		4066	709 (14.85)	Ref	
Yes	1614	260 (16.22)	−0.07 (0.683)		482	85 (19.71)	0.17 (0.446)		2096	345 (16.95)	0.05 (0.753)	
**BMI ≥ 30**				**0.58**				**0.349**				**0.673**
No	2879	397 (12.18)	Ref		1512	262 (17.17)	Ref		4391	659 (13.73)	Ref	
Yes	1441	310 (19.26)	−0.19 (0.320)		330	85 (25.74)	0.22 (0.367)		1771	395 (20.42)	−0.11 (0.453)	
**Family history of blood pressure**				**0.938**				**0.902**				**0.976**
No	2591	425 (13.2)	Ref		1281	227 (16.44)	Ref		3872	652 (14.18)	Ref	
Yes	1729	282 (16.27)	−0.05 (0.819)		561	120 (23.44)	0.05 (0.832)		2290	402 (17.89)	−0.002 (0.988)	
**Family history of diabetes**				**0.000**				**0.01**				**0.000**
No	2787	402 (10.87)	Ref		1317	219 (15.51)	Ref		4104	621 (12.26)	Ref	
Yes	1533	305 (19.7)	1.02***(0.000)		525	128 (25.53)	0.54*(0.015)		2058	433 (21.04)	0.83***(0.000)	
**Family history of total cholesterol**				**0.677**				**0.309**				**0.285**
No	3210	526 (14.55)	Ref		1542	290 (19.11)	Ref		4752	816 (15.92)	Ref	
Yes	1110	181 (14.49)	−0.05 (0.829)		300	57 (17.6)	−0.30 (0.320)		1410	238 (15.1)	−0.16 (0.366)	
**Waist-to-Hip Ratio**^**b**^				**0.001**				**0.126**				**0.000**
Normal	1365	115 (5.59)	Ref		566	82 (10.74)	Ref		1931	197 (6.88)	Ref	
Abnormal	2705	560 (20.18)	0.68**(0.002)		1256	265 (23.08)	0.33 (0.184)		3961	825 (21.02)	0.57***(0.001)	
**Hypertriglyceridemia**^**c**^				**0.002**				**0.009**				**0.000**
No	3365	481 (12.39)	Ref		1132	166 (14.41)	Ref		4497	647 (12.85)	Ref	
Yes	955	226 (22.5)	0.56**(0.004)		710	181 (25.75)	0.51*(0.014)		1665	407 (23.82)	0.57***(0.000)	
**Physical activity**				**0.755**				**0.36**				**0.943**
Insufficient	2177	386 (15.98)	Ref		668	126 (21.39)	Ref		2845	512 (17.13)	Ref	
Sufficient	2143	321 (13.51)	0.05 (0.739)		1174	221 (17.72)	0.12 (0.561)		3317	542 (14.81)	0.06 (0.659)	

*CI* Confidence interval; *p* values in parentheses, * *p* < 0.05 ** *p* < 0.01 *** *p* < 0.001; ^a^SBP ≥ 140 and/or DBP ≥ 90 and/or currently on medication; ^b^Abnormal defined as > 0.85 for female & > 0.90 for male; ^c^ Serum triglycerides ≥1.7 mmol/L

### Treatment and control status of DM

Among all the respondents with raised blood glucose or on medication for blood glucose, 83% were known cases of DM while 17% were newly diagnosed (Table 5). 14.3% of respondents were on a specific anti-diabetic medication and had a controlled glucose level (treatment success), 13.2% were on medication and a raised blood glucose level (treatment failure), and 55.5% were not taking medication (although diagnosed) and had a raised blood glucose level.

**Table 5:** Prevalence of the population with raised blood glucose or on medication for blood glucose disaggregated by diagnosis and treatment status

Category	On medication and glucose < 7 mmol/L	On medication and glucose ≥ 7 mmol/L	Not aware of raised glucose ≥ 7 mmol/L	Not taking prescribed medication and glucose ≥ 7 mmol/L	Total N
**Age group**					
18–29	12.0 (0.7–2.3)	10.3 (6.9–15.1)	39.2 (34.3–44.3)	49.2 (43.9–54.6)	87
30–39	6.6 (4.9–8.9)	6.5 (4.9–8.5)	29.1 (25.9–32.3)	57.8 (54.2–61.4)	215
40–49	12.1 (10.3–14.2)	10.1 (8.3–12.1)	16.7 (14.4–19.2)	61.1 (57.0–65.0)	259
50–59	25.3 (21.8–29.1)	11.3 (9.5–13.4)	8.8 (7.5–10.3)	54.6 (50.4–58.8)	236
60+	14.2 (12.0–16.5)	23.3 (19.9–27.1)	12.8 (10.8–15.0)	49.8 (45.2–54.4)	226
**Sex**					
Male	13.0 (11.5–14.8)	10.4 (8.9–12.2)	20.8 (19–22.7)	55.7 (52.6–58.7)	526
Female	15.7 (13.9–17.6)	16.0 (14.2–18)	13.0 (11.5–14.6)	55.3 (25.5–58.1)	497
**Nationality**					
Omani	15.3 (13.8–17.0)	14.9 (13.3–16.6)	13.4 (12.1–14.7)	56.5 (53.7–59.1)	683
Non-Omani	12.3 (10.4–14.5)	9.7 (7.9–11.7)	24.5 (22.2–26.8)	53.6 (50.6–56.6)	340
**Overall**	**14.3** (13.1–15.6)	**13.2** (12–14.5)	**17.0** (15.9–18.2)	**55.5** (53.4–57.6)	1023

Small but significantly higher prevalence was observed for Omani nationals under medication and had controlled blood glucose status compared to non-Omani residents (15.3% vs 12.3%, *p* < 0.05), but no significant difference was found between male and females in this group. A majority of newly discovered cases with raised blood glucose level was observed in the 18–29 years age group (39.2%), males (20.8%) and non-Omani residents (24.5). Similarly, a majority of respondents not currently on medication and raised blood glucose level was found among 40–49 years old (61%), and Omani nationals (56.5%).

**Fig. 1:**
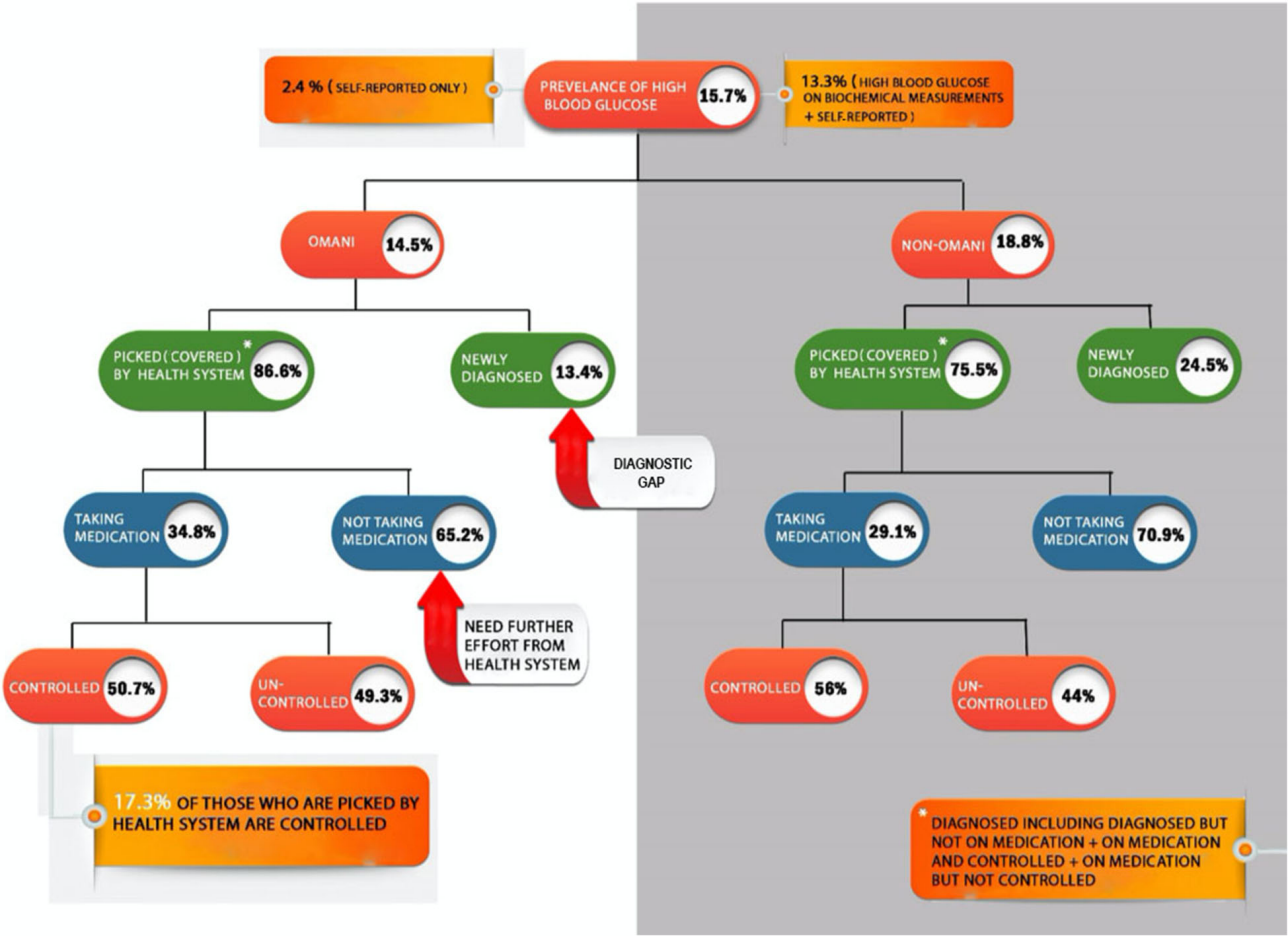
Diabetes chart by nationality, diagnosis, treatment status, and control status

Figure 1 reveals that among the 14.5% Omani nationals, 86.6% were picked by the health system and 13.4% were newly diagnosed. Of those who were covered by the health system, only 34.8% were taking the prescribed medications and 65.2% were not. Of those who were taking their prescribed medication, only 50.7% were controlled for DM. This demonstrates that only 17.3% of those who were picked by the health system had controlled blood glucose status. Similarly, for the 18.8% of non-Omani residents, 75.5% were picked by the health system and 24.5% were newly diagnosed. Of those who were covered by the health system, only 29% were taking the medications. Of those who were taking medication, only 56% were controlled for DM.

## Discussion

This study represents the largest multistage stratified community-based national survey in Oman which included all the 11 governorates of Oman. A high response rate was achieved with the employment of the standardized STEPS questionnaire, robust methodology, and adherence to STROBE guidelines [14].

The results of this survey revealed a high prevalence of DM and prediabetes among the Oman population. Some national and regional surveys were conducted previously which showed the prevalence of diabetes between 1991 and 2010, with the last main national survey conducted in 2008 [5]. An increase in the prevalence of DM was found from the 2008 national survey (12.3%) when compared to our results in 2017 (15.7%) [15]. Strikingly, there has been a sharp increase in prevalence of prediabetes from 4.4% in 2008 to 11.8% in the current study. Our results for DM were similar to other STEPS studies conducted in the WHO Eastern Mediterranean (EMR) region, but higher than the prevalence reported in other Asian countries [16–19]. Omani nationals (14.5%) were found to have a significantly lower prevalence than non-Omani residents (18.8%) in this survey. This may be attributed to the universal healthcare system which provides free primary healthcare for Omani citizens and the difference in lifestyle between the two subgroups.

It has been reported that biological composition as well as obesity has an effect on sex differences associated with the burden and complication of DM [20]. In this present study, the prevalence of diabetes and prediabetes is slighter higher (though not significant) in females than in males as opposed to other studies globally which show a higher prevalence among males as compared to females [19, 21, 22]. Our results are corroborated by higher prevalence of overweight and obesity found among females than males in the survey [8]. Our results show that one third of the population with DM are under the age of 50. Based on these results, DM did increase with age although it is not limited to older subjects. This adds to the growing body of evidence suggesting that diabetes is also evolving among the younger population as well [18, 19, 23].

Different studies showed that both or either overall obesity or central obesity was associated with conferring risk of diabetes [18, 19]. This study found that an abnormal waist-hip ratio was independently associated with diabetes which is similar to the results in most of the other studies [24–26]. Literature has already shown the protective effects of physical activity against obesity [27]. It is well known that family history of DM is a strong, independent predictor of the disease which was confirmed in our study and supported by most other studies which can be used as a public health tool [28, 29]. Hypertriglyceridemia, but not family history of total cholesterol, was significantly associated with diabetes. Constant increments in serum triglyceride levels were found to be an independent risk factor for diabetes mellitus as well as associated with increased risk for CVD [30, 31]. The study results indicate that elderly individuals, hypertensive, or those with a family history of DM comprise an notable group for screening [5, 24, 27].

The ratio of newly diagnosed to total patients with DM (17%) was found to be considerably lower as compared to the 2008 national survey [15]. This proportion comes down to 13% for Omanis and could be attributed to the national non-communicable diseases screening program for those aged 40 years and above. The DM cases with controlled glucose level have also shown a slight reduction from 2008 (17% in 2008 vs 14% in 2017). However, most concerning is the bulk of uncontrolled cases which accounts for over two-thirds of the cases with raised blood glucose, out of which around 56% are not taking any medication to control their blood glucose level although they have been diagnosed with DM. It is this pool of cases of DM left uncontrolled which would become more prone to complications, and ultimately put a higher financial strain on the country. When assessing only the Omani national population who are served by free primary healthcare, about half of those taking medication were shown to have a controlled blood glucose status. This reveals that only 17% of the individuals who were picked by the health system had a controlled blood glucose status, which is an alarming figure. Challenges of health promotion as part of Oman’s Health Vision 2050 have to be tackled to address unsafe behaviours and subsequently health-related interventions [32]. It is of paramount importance that further research looks into understanding the underlying issues to be able to address them at the root level [33].

### Study limitations

Firstly, due to the nature of the study design being cross-sectional, any casual inferences should be interpreted with caution. Secondly, blood glucose measurement was done by a spot analyser rather than venous blood glucose estimation due to logistical constraints. However, regular quality control checks on blood glucose measurement was done as per the manufacturer’s instructions. Thirdly, only fasting blood glucose level was used to measure for diabetes and prediabetes rather than the more sensitive oral glucose tolerance test (OGTT) or the HbA1c test due to financial and logistical constraints of a large-scale household community survey.

## Conclusion

In conclusion, the current study provides reliable evidence regarding the high burden of DM among the adult population in Oman. With around 28% of the general population having either diabetes or prediabetes, urgent attention is needed to address this significant burden on the health system. The newly diagnosed cases of DM in the community should not be neglected and also be offered early treatment in order to avoid complications. The reasons for uncontrolled DM in Oman warrant further research and innovative strategies to address this population. Prevention of DM should have a high priority in public health development plans in Oman to prevent associated rising economic costs.

## Data Availability

The datasets generated and/or analysed during the current study are not publicly available due to data sharing policies but are available from the corresponding author on reasonable request.

## Abbreviations

DM: Diabetes mellitus
USD: United States Dollars
GCC: Gulf Cooperation Council
WHO: World Health Organization
MENA: Middle Eastern and North African
BMI: Body Mass Index
GPAQ: Global Physical Activity Questionnaire
STROBE: Strengthening the Reporting of Observational Studies in Epidemiology
EMR: Eastern Mediterranean Region
NCD: Non-communicable Diseases

## References

[1] International Diabetes Federation (2019). IDF Diabetes Atlas.

[2] Al Slamah T, Nicholl BI, Alslail FY, Melville CA (2017). Self-management of type 2 diabetes in gulf cooperation council countries: a systematic review. PLoS One.

[3] Morgan SA, Ali MM, Channon AA, Al-Sabahi S, Al Suwaidi H, Osman N (2019). Prevalence and correlates of diabetes and its comorbidities in four gulf cooperation council countries: evidence from the world health survey plus. J Epidemiol Community Health.

[4] Institute for Health Metrics and Evaluation (2018). Oman. Global Burden of Disease Study 2017 (GBD 2017)..

[5] Al-Lawati JA, Panduranga P, Al-Shaikh HA, Morsi M, Mohsin N, Khandekar RB (2015). Epidemiology of diabetes mellitus in Oman results from two decades of research. Sultan Qaboos Univ Med J.

[6] Asfour MG, Samantray SK, Dua A, King H (1991). Diabetes mellitus in the Sultanate of Oman. Diabet Med.

[7] Majeed A, El-Sayed AA, Khoja T, Alshamsan R, Millett C, Rawaf S (2014). Diabetes in the middle-east and North Africa: an update. Diabetes Res Clin Pract.

[8] Al-Mawali A, Jayapal SK, Morsi M, Al-Shekaili W, Pinto AD, Al-Kharusi H (2020). Prevalence of Risk Factors of Non-Communicable Diseases in the Sultanate of Oman: STEPS Survey 2017.

[9] NCDs | The STEPS Instrument and Support Materials. [cited 2020 May 12]. Available from: https://www.who.int/ncds/surveillance/steps/instrument/en/.

[10] Hypertension. [cited 2020 May 12]. Available from: https://www.who.int/health-topics/hypertension/#tab=tab_1.

[11] WHO | The guideline development group for the diagnosis and pharmacological treatment of hypertension in adults. WHO. 2020. Available from: https://www.who.int/cardiovascular_diseases/guidelines/hypertension/en/

[12] Global Physical activity Questionaire Analysis. World Health Organisations. [cited 2020 May 12]. Available from: https://www.who.int/ncds/surveillance/steps/resources/GPAQ_Analysis_Guide.pdf.

[13] Global Physical Activity Questionnaire Analysis Guide GPAQ Analysis Guide Global Physical Activity Questionnaire (GPAQ) Analysis Guide.[cited 2020 May 12] Available from: https://www.who.int/ncds/surveillance/steps/resources/GPAQ_Analysis_Guide.pdf.

[14] STROBE Statement. STROBE group. 2014 [cited 2020 May 12]. Available from: https://www.strobe-statement.org/index.php?id=strobe-home

[15] Morsi M, Elaty A, Attia M, Al RA (2012). Oman world health survey: part 1 methodology, sociodemographic profile and epidemiology of non-communicable diseases in Oman. Oman Med J..

[16] Tripathy JP, Thakur JS, Jeet G, Chawla S, Jain S, Pal A (2017). Prevalence and risk factors of diabetes in a large community-based study in North India: results from a STEPS survey in Punjab, India. Diabetol Metab Syndr.

[17] World Health Organization. Noncommunicable diseases and their risk factors. STEPS Country Reports. [cited 2020 Jul 1]. Available from: who.int/ncds/surveillance/steps/reports/en.

[18] Akter S, Rahman MM, Abe SK, Sultana P (2014). Prevalence of diabetes and prediabetes and their risk factors among Bangladeshi adults: a nationwide survey. Bull World Health Organ.

[19] Pham NM, Eggleston K (2016). Prevalence and determinants of diabetes and prediabetes among Vietnamese adults. Diabetes Res Clin Pract.

[20] Peters SA, Woodward M (2018). Sex differences in the burden and complications of diabetes. Curr Diab Rep.

[21] Msyamboza KP, Mvula CJ, Kathyola D (2014). Prevalence and correlates of diabetes mellitus in Malawi: population-based national NCD STEPS survey. BMC Endocr Disord.

[22] Gaio V, Antunes L, Namorado S, Barreto M, Gil A, Kyslaya I (2018). Prevalence of overweight and obesity in Portugal: results from the first Portuguese health examination survey (INSEF 2015). Obes Res Clin Pract.

[23] Hilawe EH, Chiang C, Yatsuya H, Wang C, Ikerdeu E, Honjo K (2016). Prevalence and predictors of prediabetes and diabetes among adults in Palau: population-based national STEPS survey. Nagoya J Med Sci.

[24] Al-Lawati JA, Jousilahti PJ (2004). Prevalence and 10-year secular trend of obesity in Oman. Saudi Med J..

[25] Al-Riyami AA, Afifi MM (2003). Prevalence and correlates of obesity and central obesity among Omani adults. Saudi Med J.

[26] Balhareth A, Meertens R, Kremers S, Sleddens E (2019). Overweight and obesity among adults in the Gulf States: A systematic literature review of correlates of weight, weight-related behaviours, and interventions. Obesity Rev. Blackwell Publishing Ltd.

[27] Mabry R, Owen N, Eakin E (2014). A National Strategy for promoting physical activity in Oman: a call for action. Sultan Qaboos Univ Med J.

[28] Hariri S, Yoon PW, Qureshi N, Valdez R, Scheuner MT, Khoury MJ (2006). Family history of type 2 diabetes: a population-based screening tool for prevention?. Genet Med.

[29] Consortium I (2013). Others. The link between family history and risk of type 2 diabetes is not explained by anthropometric, lifestyle or genetic risk factors: the EPIC-InterAct study. Diabetologia..

[30] Ye X, Kong W, Zafar MI, Chen L-L (2019). Serum triglycerides as a risk factor for cardiovascular diseases in type 2 diabetes mellitus: a systematic review and meta-analysis of prospective studies. Cardiovasc Diabetol.

[31] Beshara A, Cohen E, Goldberg E, Lilos P, Garty M, Krause I (2016). Triglyceride levels and risk of type 2 diabetes mellitus: a longitudinal large study. J Investig Med.

[32] Al Hinai H, Al Mufarji K, Al Siyabi H, Al Anqoudi Z, Al Saadi R, Al Awaidy S. Health promotion strategy as part of Vision 2050 in Oman: the way forward. Global Health Promotion. 2020;27(4):145–9. 10.1177/1757975920909115. 10.1177/1757975920909115 32167017

[33] Al Mawali AHN, Al Qasmi AM, Al Sabahi SMS, Idikula J, Abd Elaty MA, Morsi M (2017). Oman vision 2050 for health research: a strategic plan for the future based on the past and present experience. Oman Med J.

